# Early-Life Stress Modulates Gut Microbiota and Peripheral and Central Inflammation in a Sex-Dependent Manner

**DOI:** 10.3390/ijms22041899

**Published:** 2021-02-14

**Authors:** Hae Jeong Park, Sang A. Kim, Won Sub Kang, Jong Woo Kim

**Affiliations:** 1 Department of Pharmacology, School of Medicine, Kyung Hee University, Seoul 02447, Korea; hjpark17@khu.ac.kr (H.J.P.); sanga0568@naver.com (S.A.K.); 2 Department of Neuropsychiatry, School of Medicine, Kyung Hee University, Seoul 02447, Korea; menuhinwskang@khu.ac.kr

**Keywords:** early life stress, maternal separation, gut microbiota, inflammation, kynurenine, cytokine, high-throughput sequencing, hippocampus, anxiety-like behavior

## Abstract

Recent studies have reported that changes in gut microbiota composition could induce neuropsychiatric problems. In this study, we investigated alterations in gut microbiota induced by early-life stress (ELS) in rats subjected to maternal separation (MS; 6 h a day, postnatal days (PNDs) 1–21), along with changes in inflammatory cytokines and tryptophan-kynurenine (TRP-KYN) metabolism, and assessed the differences between sexes. High-throughput sequencing of the bacterial 16S rRNA gene showed that the relative abundance of the *Bacteroides* genus was increased and that of the Lachnospiraceae family was decreased in the feces of MS rats of both sexes (PND 56). By comparison, MS increased the relative abundance of the *Streptococcus* genus and decreased that of the *Staphylococcus* genus only in males, whereas the abundance of the *Sporobacter* genus was enhanced and that of the *Mucispirillum* genus was reduced by MS only in females. In addition, the levels of proinflammatory cytokines were increased in the colons (IFN-γ and IL-6) and sera (IL-1β) of the male MS rats, together with the elevation of the KYN/TRP ratio in the sera, but not in females. In the hippocampus, MS elevated the level of IL-1β and the KYN/TRP ratio in both male and female rats. These results indicate that MS induces peripheral and central inflammation and TRP-KYN metabolism in a sex-dependent manner, together with sex-specific changes in gut microbes.

## 1. Introduction

Early-life stress (ELS) such as maternal neglect, social isolation or abuse during childhood is associated with an increased risk of psychiatric problems such as cognitive deficits, anxiety disorders and depression, persisting into adulthood [1,2]. Various rodent models of ELS including rats subjected to maternal separation (MS) have been used to study the psychopathology of anxiety disorders and depression [3,4,5]. Indeed, previous experimental studies have shown alterations of neurodevelopmental, neuroendocrinal and neuroinflammatory factors such as corticotropin-releasing hormone (CRH), interleukin-1β (IL-1β) and brain-derived neurotrophic factor (BDNF) in MS rodents, along with increased anxiety- and depression-related behaviors [6,7,8]. Pups exposed to MS have also shown to develop an increased threshold to pain, possibly related to stress [9].

Emerging evidence has suggested that the gut microbiota can modify brain development, mood and behavior, modulating neuroendocrine and neuroimmune systems [10,11,12]; in particular, recent studies have reported the modulation of the gut microbiota and inflammation in depression and anxiety [13,14]. Pusceddu et al. [15] revealed that *Flexibacter* and *Prevotella*, which have reported to be associated with colitis, were more abundant in the gut of MS rats. Wong et al. [13] showed that the inhibition of caspase-1, an inflammasome factor, rebalanced stress-induced gut microbiota changes and attenuated stress-induced anxiety- and depression-like behaviors and reducing inflammation. Zhang et al. [16] reported that the blockade of interleukin-6 (IL-6) through treatment with an IL-6 receptor antibody exerted antidepressant effects in mice subjected to social defeat, restoring the altered gut microbiota composition. In addition, probiotics such as *Lactobacillus mucosae*, *Bifidobacterium longum* and *Faecalibacterium prausnitzii* have been shown to suppress anxiety- and depression-like behaviors, microglial activation and the upregulation of inflammatory cytokines induced by chronic stress [17,18].

In rats or mice exposed to stress, the perturbation of gut microbiota and inflammation can also modulate the metabolism of tryptophan (TRP) to kynurenine (KYN) or serotonin (5-hydroxytryptamine; 5-HT) [19,20,21]. Inflammatory cytokines such as interferon-γ (IFN-γ) and IL-6 activate the production of indoleamine-2,3-dioxygenases (Idos), which metabolize TRP to KYN, augmenting KYN production and attenuating 5-HT levels [19]. An increase in KYN/TRP ratios has accordingly been shown in depression-like rats, together with the elevation of proinflammatory cytokines and alterations in gut microbiota [21].

Sex-based differences in the regulation of neurotransmission, the activation of the immune system and the incidence of psychiatric diseases have been demonstrated [12,22,23]. Previous studies suggested that sex-specific alterations in the gut microbiota could influence these sex-based differences [12,24]. In rodents exposed to prenatal stress or multihit early adversity combining prenatal and postnatal stress, males showed greater emotional vulnerability and alterations of gut microbiota than females [25,26]. In addition, Audet [27] reported that sex-based differences in the development of the gut microbiome–immune–brain axis could contribute to the sex-specific pathogenesis of depression and anxiety, leading to distinct gut bacterial communities, immune-signaling pathways and neuroinflammatory processes in adult males and females.

Considering these previous reports, we speculated that ELS would alter gut microbiota composition in a sex-specific manner, leading to the sex-specific pathophysiology of psychiatric disorders via distinct inflammation processes in males and females. In previous studies, sex-specific inflammatory responses have been reported in ELS rodents subjected to MS [28,29]; however, to the best of our knowledge, there is not any report on sex-specific alterations in gut microbiota composition or associations of those with sex-specific inflammatory responses in ELS models, yet. In order to determine the sex-specific pathophysiology of psychiatric disorders induced by ELS, we investigated the alterations of gut microbiota composition, inflammatory cytokine levels and TRP-KYN metabolism in rats subjected to MS during postnatal days (PNDs) 1–21 and assessed the differences between males and females.

## 2. Results

### 2.1. Effect of MS on Anxiety-Like Behavior

**Figure 1:**
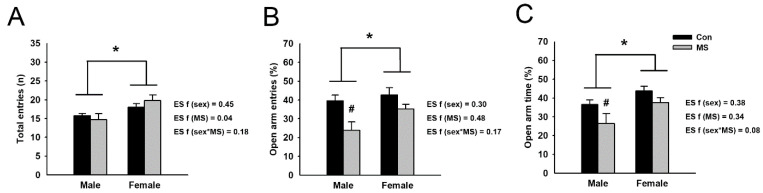
Anxiety-like behavior in maternal separation (MS) rats. Anxiety-like behavior was examined using the elevated plus-maze (EPM) test. (**A**) The number of total entries was assessed in control and MS rats. Anxiety-related behavior is indicated by the frequencies of open arm entries (**B**) and time spent in the open arms (**C**). Data are expressed as mean ± SEM. A two-way ANOVA test, * *p* < 0.05 on male vs. female; ^#^ *p* < 0.05 on control vs. MS within each sex. Effect size (ES) f was calculated using G*Power 3. *n* = 10–12 per group.

To identify the anxiety-like behavior induced by MS, rats were tested on an elevated plus maze (EPM). A two-way ANOVA revealed no interaction between MS and sex in the number of total entries, the frequency of open arm entries and the time spent in open arms. However, significant increases in the number of total entries (F_3,42_ = 9.089, *p* = 0.004), the frequency of open arm entries (F_3,42_ = 4.118, *p* = 0.049) and the spent time in open arms (F_3,42_ = 6.593, *p* = 0.014) were observed in females compared to males. In addition, significant reductions in the frequency of open arm entries (F_3,42_ = 10.436, *p* = 0.002) and the spent time in open arms (F_3,42_ = 5.223, *p* = 0.027) were found in MS rats compared to control rats (Figure 1). Analyzing the effect of MS within each sex using a nested-design two-way analysis showed that MS significantly decreased the frequency of open arm entries (*p* = 0.003) and the spent time in open arms (*p* = 0.046) for male rats, but not for females.

### 2.2. Sex-Specific Alterations of Gut Microbiota Composition in MS Rats

**Figure 2:**
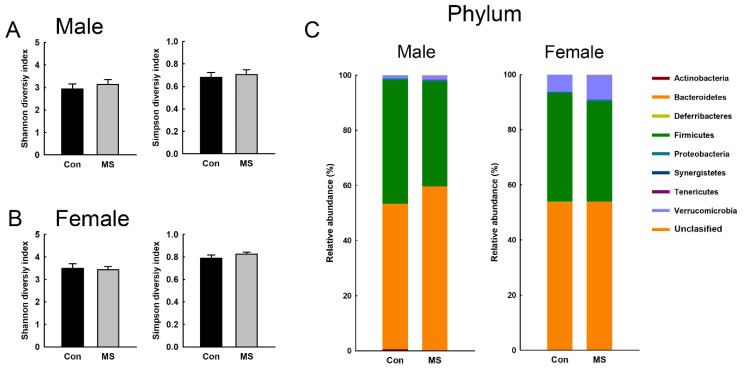
Effect of maternal separation (MS) on fecal microbiota composition at the phylum level. Alpha diversity (Shannon and inversed Simpson indices) in male (**A**) and female rats (**B**). Data are mean ± SEM. (**C**) Mean microbial composition at the phylum level. *n* = 7 per group.

**Figure 3:**
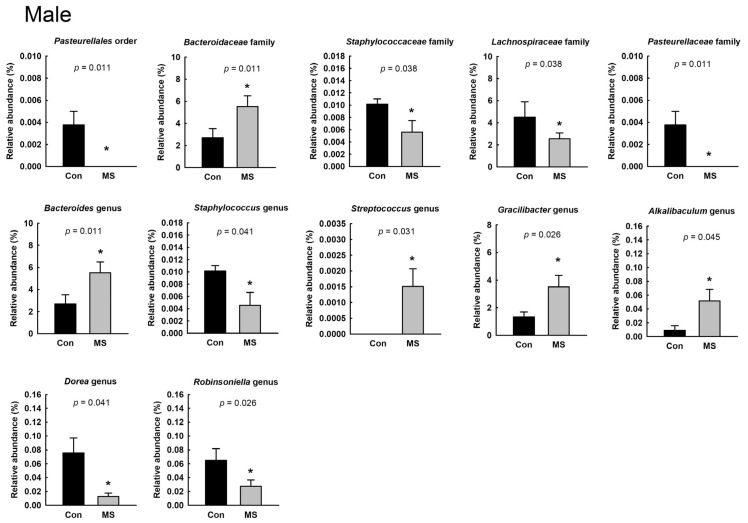
Effect of maternal separation (MS) on fecal microbiota composition in male rats. Taxa whose relative abundances are significantly changed by MS. Data are mean ± SEM. The abundance of *Streptococcus* in control rats and of Pasteurellales and Pasteurellaceae in MS rats was not detected. Wilcoxon rank-sum test, * *p* < 0.05. *n* = 7 per group.

**Figure 4:**
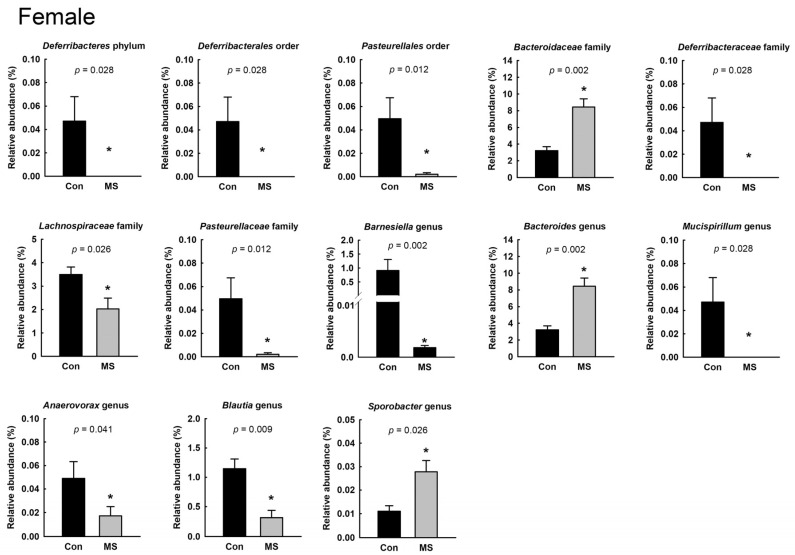
Effect of maternal separation (MS) on fecal microbiota composition in female rats. Taxa whose relative abundances are significantly changed by MS. Data are mean ± SEM. The abundance of Deferribacteres, Deferribacterales, Deferribacteraceae and *Mucispirillum* was not detected in MS rats. Wilcoxon rank-sum test, * *p* < 0.05. *n* = 6 per group.

To characterize the gut microbiota composition, 6,366,824 reads were generated from 26 fecal pellets. A total of 1,444,514 reads remained after quality filtering, with a mean of 53,500 reads per sample. The microbial diversity within a sample did not differ between the control and MS rats, for both males and females (Figure 2A,B). Figure 2C shows the relative abundance at the phylum level in the control and MS rats. The relative abundance of the Deferribacteres phylum was significantly different between female control and MS rats. In female control rats, the abundance of Deferribacteres was 0.05% ± 0.02% whereas that was not detected in any female rat exposed to MS. In both males and females, we found significant changes in the relative abundance of the Pasteurellales order; Bacteroidaceae, Lachnospiraceae and Pasteurellaceae families; and *Bacteroides* genus (Figure 3 and Figure 4; *p* < 0.05). The relative abundance of the Pasteurellales order and Lachnospiraceae and Pasteurellaceae families were decreased in both male and female MS rats, whereas the abundance of the *Bacteroides* genus together with that of the Bacteroidaceae family was increased. Among the genera belonging to the Lachnospiraceae family, in male MS rats, the abundance of the *Dorea* and *Robinsoniella* genera was significantly reduced, while in female MS rats, that of the *Blautia* genus was diminished. Moreover, we observed sex-based differences in the changes in microbiota composition induced by MS. In males, MS decreased the relative abundance of the *Staphylococcus* genus along with the Staphylococcaceae family and increased that of the *Streptococcus*, *Gracilibactoer* and *Alkalibaculum* genera (Figure 3). In females, MS reduced the relative abundance of the *Barnesiella*, *Anaerovorax*, and *Mucispirillum* genera belonging to the Deferribacteres phylum, Deferribacterales order and Deferribacteraceae family. By contrast, that of *Sporobacter* genus was elevated in female MS rats (Figure 4). However, the differences were not significant after multiple-comparison correction by the Benjamini–Hochberg algorithm.

### 2.3. Increase in IL-1β in the Sera of Male, but Not Female, MS Rats

We examined the cytokine levels in the sera of the MS rats (Table 1). We found that the level of IL-1β was significantly increased in male MS rats, compared to male control rats (adjusted *p* = 0.027). However, in female rats, no statistical significance in cytokine levels was detected between control and MS rats.

### 2.4. Increase in Proinflammatory Cytokines and Decrease in Zonula Occludens-1 (Zo-1) Expression in the Colons of Male, but Not Female, MS Rats

In order to determine MS-induced colonic inflammation, cytokine levels were examined in the colons of the control and MS rats. The colonic cytokine levels are shown in Table 1. MS significantly elevated the levels of IFN-γ (adjusted *p* < 0.001), and IL-6 (adjusted *p* = 0.029) in male rats. However, in females, any significant difference in cytokine levels was not observed between control and MS.

**Figure 5:**
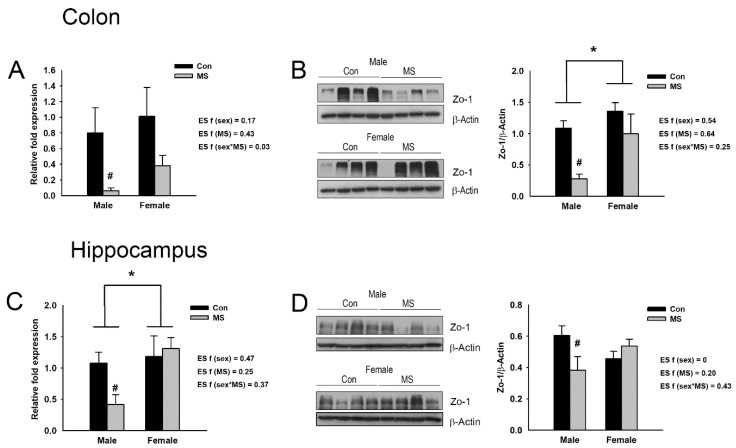
Effect of maternal separation (MS) on the expression levels of tight junction protein Zonula occludens-1 (Zo-1) and gene encoding it. (**A**) The mRNA and (**B**) protein expression of Zo-1 in the distal colon of male and female MS rats. (**C**) The mRNA and (**D**) protein expression of Zo-1 in the hippocampus of male and female MS rats. The mRNA and protein expression levels are normalized against Gapdh and β-Actin, respectively. Data are mean ± SEM. A two-way ANOVA test, * *p* < 0.05 on male vs. female; ^#^ *p* < 0.05 on control vs. MS within each sex. Effect size (ES) f was calculated using G*Power 3. *n* = 6–10 per group.

Tight junction proteins are essential for the maintenance of intestinal barrier function, connecting intestinal epithelial cells and regulating paracellular permeability. Disruption of intestinal barrier function, which results in increased intestinal permeability and in turn, facilitates translocation of harmful substances and pathogens to the bloodstream, has been associated with various injury events, including microbial degradation and exposures to bacterial toxin and proinflammatory cytokines [30]. In order to determine the disruption of intestinal barrier function in MS rats, we examined the levels of mRNAs for genes encoding tight junction proteins such as occludin (Ocln), claudin 1-5 (Cldn 1-5) and Zo-1 in the colons of MS rats (Figure S1). A two-way ANOVA revealed that MS significantly reduced the mRNA expression of Zo-1 in the colon (F_3,36_ = 7.250, *p* = 0.011; Figure 5A), but not those of Ocln and Cldn 1-5 (Figure S1). In particular, the analysis of the effect of MS within each sex showed that the mRNA expression of Zo-1 was significantly reduced in the colons of male MS rats (*p* = 0.047) but not females (Figure 5A). The protein level of Zo-1 was also assessed in the colons of the MS rats (Figure 5B). A two-way ANOVA showed a significant sex effect; the level of Zo-1 was higher in females than in males (F_3,20_ = 6.972, *p* = 0.016). A significant MS effect was also observed; MS decreased the protein level of Zo-1 in the colon (F_3,20_ = 9.856, *p* = 0.005). Especially, analyzing the effect of MS within each sex showed that the Zo-1 expression was significantly reduced in the colons of male MS rats (*p* = 0.006) but not females.

### 2.5. Increase in Proinflammatory Cytokines and Decrease in Zo-1 Expression in the Hippocampi of MS Rats

The cytokine levels in the hippocampus showed in Table 1. In males, the level of IL-1β was higher in MS rat than control rats (adjusted *p* = 0.048), whereas that of IL-4 was lower in MS rats than control rats (adjusted *p* < 0.001). In females, we found that MS increased the levels of IL-1β (*p* = 0.042) and IL-2 (*p* = 0.025). However, the statistical significance did not remain after multiple-comparison correction using the Benjamini–Hochberg method.

We also examined the mRNA and protein expression of Zo-1 in the hippocampus. A significant sex effect on mRNA expression was detected (F_3,20_ = 5.219, *p* = 0.033); the expression was increased in females compared to males. On the protein level, a significant interaction effect between MS and sex (F_3,28_ = 5.925, *p* = 0.022) was observed (Figure 5C,D). The analysis of the effect of MS within each sex indicated that both the mRNA and protein expression of Zo-1 were only reduced in the hippocampi of the male rats (*p* = 0.044 and *p* = 0.018, respectively) (Figure 5C,D).

### 2.6. Sex-Specific Activation of TRP-KYN Metabolic Pathway in MS Rats

**Figure 6:**
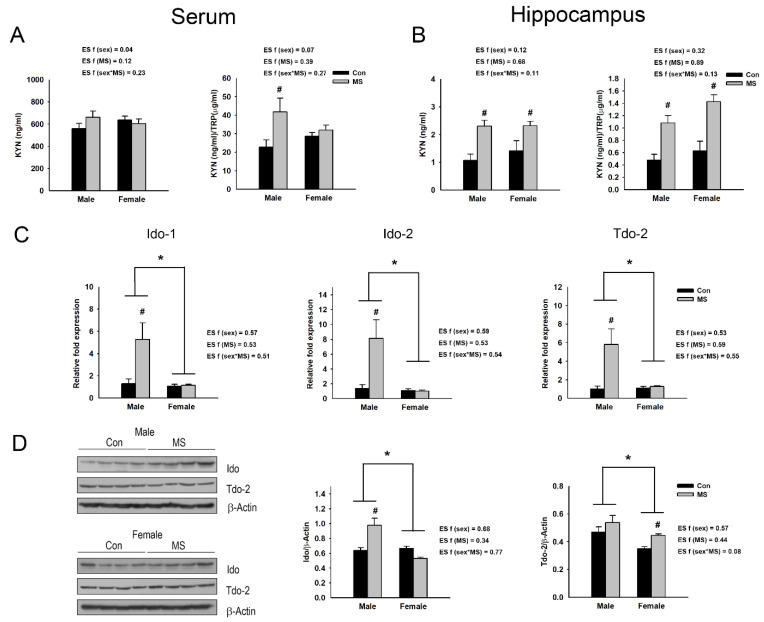
Effect of maternal separation (MS) on kynurenine (KYN) pathway. Kynurenine level and KYN/tryptophan (TRP) ratio are assessed in the serum (**A**) and the hippocampus (**B**) of MS rats. (**C**) The mRNA levels and (**D**) protein of indoleamine-2,3-dioxygenase-1/-2 (Ido-1/-2) and tryptophan 2,3-dioxygenase-2 (Tdo-2) in the hippocampus of male and female MS rats. Data are expressed as mean ± SEM. A two-way ANOVA test, * *p* < 0.05 on male vs. female; ^#^ *p* < 0.05 on control vs. MS within each sex. Effect size (ES) f was calculated using G*Power 3. *n* = 6–10 per group.

Increased levels of proinflammatory cytokines can affect Ido-1/2 and tryptophan 2,3-dioxygenase-2 (Tdo-2) activity, promoting the metabolism of TRP to KYN [19,31,32]. We assessed the KYN/TRP ratio in the sera and hippocampi of MS rats. No significant interaction effect between sex and MS on the serum KYN level and KYN/TRP ratio was detected. However, MS significantly elevated the KYN/TRP ratio in the serum (F_3,36_ = 6.101, *p* = 0.018) but not the level of KYN. The analysis of the effect of MS within each sex revealed a significant increase in the KYN/TRP ratio in males (*p* = 0.005) but not females (Figure 6A).

In the hippocampus, no significant interaction effect between sex and MS on the KYN level and KYN/TRP ratio was observed. However, the levels of KYN (F_3,36_ = 18.555, *p* < 0.001) and KYN/TRP ratios (F_3,36_ = 32.151, *p* < 0.001) were significantly higher in the hippocampi of the MS rats than controls. By analyzing the effect of MS within each sex, it was found that MS increased the levels of KYN and KYN/TRP ratios in both male (*p* = 0.001 for both) and female rats (*p* = 0.014 and *p* < 0.001, respectively) (Figure 6B). Additionally, a two-way ANOVA revealed significant interaction effects between sex and MS on the mRNA expression of Ido-1/2 and Tdo-2 (F_3,20_ = 6.326, *p* = 0.021 for Ido-1; F_3,20_ = 7.051, *p* = 0.015 for Ido-2; F_3,20_ = 7.318, *p* = 0.014 for Tdo-2) and the protein expression of Ido (F_3,28_ = 18.787, *p* < 0.001) in the hippocampus. Significant sex effects on the mRNA expression of Ido-1/2 and Tdo-2 (F_3,20_ = 7.770, *p* = 0.011 for Ido-1; F_3,20_ = 8.280, *p* = 0.009 for Ido-2; F_3,20_ = 6.800, *p* = 0.017 for Tdo-2) and the protein expression of Ido (F_3,28_ = 14.599, *p* = 0.001) and Tdo-2 (F_3,28_ = 10.241, *p* = 0.003) were also detected, with higher levels in males (Figure 6C,D). MS also significantly increased the mRNA levels of Ido-1/2 and Tdo-2 (F_3,20_ = 6.787, *p* = 0.017 for Ido-1; F_3,20_ = 6.692, *p* = 0.018 for Ido-2; F_3,20_ = 6.800, *p* = 0.009 for Tdo-2), and the protein level of Tdo-2 (F_3,28_ = 6.166, *p* = 0.019). Upon analyzing the effect of MS within each sex, MS was shown to upregulate the mRNA expression of Ido-1 (*p* = 0.021), Ido-2 (*p* = 0.001) and Tdo-2 (*p* = 0.001) and the protein expression of Ido-1 (*p* < 0.001) in male rats (Figure 6C,D); in female rats, it increased Tdo-2 at the protein level (*p* = 0.047; Figure 6D).

## 3. Discussion

In the present study, we found sex-specific effects of MS. An EPM test showed that MS induced anxiety-like behavior in male rats, decreasing the frequency of open arm entries and the time spent in open arms. Although the decreased frequency of open arm entries and time spent in open arms were also observed in female MS rats, a statistical difference was not detected. MS also changed the microbiota composition in a sex-specific manner. The levels of proinflammatory cytokines were increased in the colons and sera of male MS rats, but not females, along with elevated serum KYN/TRP ratios. In the hippocampus, increases in proinflammatory cytokines, including Ido-1/2 and/or Tdo-2, and KYN/TRP ratios were shown in both male and female MS rats, being more pronounced in males.

In the analysis of gut microbiota composition, we found that the relative abundance of the Bacteroidaceae family and *Bacteroides* genus were increased, while that of the Lachnospiraceae family was decreased, in both male and female MS rats. The Gram-negative *Bacteroides* genus can produce the endotoxin lipopolysaccharide, which induces local and/or systemic inflammation and is associated with inflammatory diseases [33,34,35,36]. Previous studies have reported the alteration of the *Bacteroides* genus in psychiatric disorders [37,38,39]. In clinical studies, the *Bacteroides* genus was found to be elevated in male patients with major depression disorder (MDD) [37] and female subjects with high depression, anxiety and stress scale (DASS-42) scores [38]. In addition, an experimental study showed that an increased abundance of the *Bacteroides* genus was associated with increased gut permeability and colonic proinflammatory states in both male and female mouse models of autism spectrum disorders (ASD) [39].

We also found an attenuation of the relative abundance of Lachnospiraceae in both male and female MS rats. A clinical study analyzing the feces of patients diagnosed with depression showed a reduction in the Lachnospiraceae family together with an increase in the Bacteroidales order [40]. Experimental studies have reported that an increase in the relative abundance of Lachnospiraceae is associated with anti-inflammatory and antidepressive effects, ameliorating gut inflammation and/or depression-like behaviors [13,41,42]. Among the microbes belonging to the Lachnospiraceae family, the *Dorea* and *Robinsoniella* genera in male MS rats and the *Blautia* genus in females were shown to be reduced in abundance. Taylor et al. [38] reported that the *Dorea* (with depression and anxiety) and *Blautia* (with anxiety) genera were negatively associated with DASS-42 scores in males. However, in their study [38], in females, any genus belonging to the Lachnospiraceae family was not associated with DASS-42 scores. Taylor et al. [38] analyzed the associations with DASS-42 scores in subjects without physician-diagnosed mood disorders; thus, the lack of association in females may have been due to subjects with relatively mild emotional symptoms. Therefore, an elevation in the relative abundance of the *Bacteroides* genus and reduction in the abundance of the Lachnospiraceae family may be involved in the induction of the anxiety-like behavior and inflammation in MS rats of both sexes.

However, we found that the relative abundance of the *Sporobacter* genus was increased and the abundance of the *Mucispirillum* genus was decreased in female MS rats. Although the function of the *Sporobacter* genus is not well known, its lower abundance has been reported in patients with immune-mediated inflammatory diseases including Crohn’s disease, ulcerative colitis and multiple sclerosis [43]. By contrast, the *Mucispirillum* genus, which inhabits the mucus layer in the colon, is reportedly related to intestinal inflammation [44,45]. In particular, *Mucispirillum* has been suggested, as a microbial marker in active colitis, to degrade mucin [46,47]. Considering these previous reports, in the colons of female MS rats, the proinflammatory response may not be induced. In addition, we observed that colonic Zo-1 expression was not changed by MS in female rats. These results indicate that inflammation and disruption of intestinal barrier associated with inflammation may not be mediated in the colon of female MS rats, although the relative abundance of the *Bacteroides* genus was enhanced and that of the Lachnospiraceae family was reduced in those rats.

By comparison, in male MS rats, an increase in the relative abundance of the *Streptococcus* genus and decrease in the abundance of the *Staphylococcus* genus was shown. The *Streptococcus* genus along with the *Bacteroides* genus has been shown to induce colitis in experimental animals [36]. The *Staphylococcus* genus has reported to promote the growth of anaerobic bacteria and maturated the neonatal gut microbiota [48,49]. Thus, Alkanani et al. [50] suggested that a reduction in *Staphylococcus* in type 1 diabetes might be associated with the limited ability of the patients to defend against the proinflammatory responses. Therefore, the elevation of *Streptococcus* and the reduction of *Staphylococcus* induced by MS may promote inflammation in males, together with Lachnospiraceae and *Bacteroides*.

Indeed, we observed more severe inflammation in males exposed to MS. In male MS rats, proinflammatory cytokines were increased in the serum (IL-1β), colon (IFN-γ and IL-6) and hippocampus (IL-1β), whereas in females, those were elevated only in the hippocampus (IL-1β and IL-2 considering raw *p*-value). Numerous clinical studies have also shown that the increased circulation levels of inflammatory markers such as IL-6 and/or CRP are associated with depression only in males or more strongly in males than females [51,52,53]. However, other studies have reported that the concentrations of IL-1β, IL-6 and/or CRP in the serum or cerebrospinal fluid were positively correlated with depression severity only, or more profoundly in females [54,55]. In addition, a post-mortem study revealed that the expression of the anti-inflammatory cytokines IL-13 and IL-4 was increased in the orbitofrontal cortices of female and male suicide victims, who had been diagnosed with depression [56]. In clinical studies, these inconsistencies may be due to a number of confounding factors that could affect cytokine levels including the duration and recurrence frequency of depression, aging, BMI, smoking, alcohol consumption and medication [57,58]. Even though there may be various confounding factors, such as the duration of MS and aging, in experimental studies, relatively more severe inflammatory responses have been exhibited in males subjected to MS than females [28,29]. In the hippocampi, prefrontal cortices and/or striatums of MS rats, the elevations of proinflammatory cytokines such as IL-6 and/or TNF-α have been revealed in both males and females, being more pronounced in males [29]. MS has also been shown to enhance the mRNA levels of TNF-α and TNF receptor-1 in the prefrontal cortices and nucleus accumbens of males, but not females [28]. Moreover, the circulating level of anti-inflammatory cytokine IL-10 was decreased in male MS rats, but not females [59]. Our results also showed more severe inflammation in males exposed to MS than in females; MS induced hippocampal inflammation in females but systemic inflammation in males, enhancing proinflammatory cytokines in the serum, colon and hippocampus and reducing anti-inflammatory cytokine IL-4 in the hippocampus, along with anxiety-like behavior.

These results are also supported by the KYN/TRP ratios. Increased KYN/TRP ratios were found in the sera of MS male, but not female, rats and in the hippocampi of both males and females. We also observed that the expression of Ido-1/2 and Tdo-2, activated by proinflammatory cytokines such as IFN-γ and IL-1β [31,32,60] was increased in the hippocampi of MS rats of both sexes. These results indicate that the elevation of Ido-1/2 and Tdo-2 by inflammatory cytokines within the hippocampus promoted the metabolism of TRP to KYN in both male and female MS rats. KYN can be further metabolized to quinolinic acid (QUIN) and kynurenic acid (KYNA), which have been shown to regulate brain homeostasis and modulate neurotransmitter systems [61]. KYNA has been suggested to be a neuroprotector and glutamate receptor antagonist, whereas QUIN has been shown to be neurotoxic and a glutamate receptor agonist [61]. In depression, the increased production of QUIN has been revealed [62,63], and the treatment of indole-pyruvic acid transformed in vivo to KYNA showed an antidepressant property in depressive rats [64]. We found that the mRNA level of kynurenine 3-monooxygenase (Kmo), which synthesizes QUIN from KYN, was increased in the hippocampi of both male and female MS rats, whereas the level of kynurenine aminotransferase (Kat), which metabolizes KYN to KYNA, was not changed (Figure S2). Thus, we expected that the produced KYN would be metabolized to QUIN rather than KYNA. The anxiety-like behavior may be induced via the elevated production of QUIN in the hippocampi of both male and female MS rats.

However, interestingly, there may be sex-specific synthesis and/or processing of KYN within the hippocampus. According to our results, Zo-1 expression was reduced in the hippocampi of male MS rats but not females. This supports the possibility that peripheral IL-1β may also facilitate hippocampal KYN synthesis in male MS rats, through increased blood–brain barrier permeability. In addition, the transport of peripheral KYN into the brain, encouraged by elevated peripheral KYN production [65], may contribute to the increase in the KYN/TRP ratio in the hippocampi of male MS rats. Moreover, considering the reduced Zo-1 levels in the colons of male MS rats, colonic IFN-γ and IL-6 may be implicated in the increase in the KYN/TRP ratios in the sera and in the hippocampi of male MS rats, through enhanced intestinal permeability.

In early infancy, increased *Lactobacillus* and *Bifidobacterium* genera and thereafter elevated Firmicutes and reduced Bacteroidetes phyla are more often shown in females [66,67,68]. It has been demonstrated that this colonization can improve nutrient availability and gut barrier functions, and reduce inflammation in females, leading to the lower susceptibility of females to environmental insults in early life [69]. Our results show that ELS augmented systemic inflammation, KYN production and severe anxiety-like behaviors more so in males than females. These findings may indicate sex-specific pathophysiology in depression and anxiety induced by ELS. In females, inflammation in the CNS may play a pivotal role in ELS-induced depression and anxiety, while in males, peripheral inflammation resulting from the alteration of the gut microbiota composition in addition to inflammation in the CNS may be involved in the development of ELS-induced depression and anxiety.

There were some limitations to our study. In the analysis of the gut microbiota composition, the differences between the control and MS rats were not shown to be significant after multiple comparison correction. In addition, the estimated effective sample size for our study was 52 (assuming that effect size = 0.4, α = 0.05 and power = 0.8); thus, our study was performed using a relatively small sample size. The results need to be validated in further replication studies with larger sample sizes.

## 4. Materials and Methods

### 4.1. Animals and Maternal Separation

Pregnant Sprague–Dawley rats (gestation day 14) were purchased from Central Lab. Animal Inc. (Seoul, Korea). The rats were individually housed with a 12 h light/dark cycle and a standard temperature (22 ± 2 °C) with food and water freely available. Neonatal maternal separation was carried out based on previous studies [70,71]. The day of delivery was designated as PND 0. After birth, litters were randomly allocated to the control or maternal separation (MS) groups. A total of 46 pups were assigned to four groups: Con-males (*n* = 12), MS-males (*n* = 12), Con-females (*n* = 12) and MS-females (*n* = 10). During PNDs 1–21, the MS pups were separated twice a day, for 180 min each time (for a total of 360 min per day), from their dam nest (09:00–12:00 a.m. and 1:00–4:00 p.m.) and placed into another clean cage with a heating pad (30 ± 2 °C). After each 180 min, the pups were returned to their dams until the next period of separation. The control pups remained in their home cage with their dams. On PND 22, all the pups were weaned and separated by sex (2–3 rats per cage) until PND 56. All the animal experiments were conducted in accordance with the animal care guidelines of the National Institute for Health (NIH) Guide, and approved by the Animal Care and Use Committee at Kyung Hee University (KHUASP-17-156).

### 4.2. EPM Test

On PND 56, the EPM test was performed as described in our previous study [72]. The EPM was made of black wood, consisted of two open arms (50 in length and 10 cm in width) and two enclosed arms (50 in length, 10 cm in width and 30 cm in wall, without a roof) and was elevated to a height of 50 cm. After being habituated to the testing room for at least 20 min, each rat was placed into the center of a maze facing one of the open arms. The time spent in and numbers of entries into the arms were recorded for 5 min. The maze was cleaned with 70% ethanol after each test.

### 4.3. Sample Collection

All of the rats were sacrificed after the EPM test on PND 56. Before they were anesthetized, feces were collected and stored at −80 °C for microbiota composition analysis. The hippocampus and segment of the distal colon (approximately 1.5 cm) from each rat were dissected out and stored at −80 °C until analysis. Blood samples were collected from each rat and then centrifuged at 3000× *g* for 10 min; the supernatants were immediately divided into aliquots, and the serum was frozen at −80 °C until analysis.

### 4.4. Microbiota Composition Analysis

Microbiota DNA was extracted from the fecal samples using a QIAamp Fast DNA Stool Mini kit (Qiagen, Hilden, Germany); the DNA concentration and quality were checked using a NanoDrop (Thermo Fisher Scientific, Waltham, MA, USA).

The V3–V4 region of the 16S rRNA gene was amplified from the extracted DNA and then sequenced on an Illumina HiSeq. After sequencing, the reads were merged using FLASH v. 1.2.11 [73]. The reads with length <400-bp and ambiguous and chimeric sequences were removed. The remaining reads were clustered into operational taxonomic units (OTUs) based on a 97% similarity threshold using QIIME v. 1.9. Taxonomy was assigned to the selected OTUs using BLASTN v. 2.2.25 based on the National Center for Biotechnology Information 16S RefSeq database. Taxonomic assignments according to the best BLASTN hit with a query coverage of the original query sequence <85% or a percent identity <85% were excluded. Estimates of the microbiota richness and diversity within a sample were calculated using the Shannon and inversed Simpson indices, including the rarefaction curve and Chao1 index.

In order to determine the effects of MS in each sex, the relative abundance of taxa was compared by the Wilcoxon rank-sum test using R v. 3.1.2. Taxa with *p*-values <0.05 were considered to be statistically significantly different. The *p* values adjusted according to the false discovery rate (FDR) using the Benjamini–Hochberg algorithm for multiple-comparison correction were also calculated.

### 4.5. Multiplex Cytokine Measurement

The levels of the inflammatory cytokines IFN-γ, IL-1α, IL-1β, IL-2, IL-4, IL-6, IL-10 and IL-13 were analyzed in the serum, distal colon and hippocampus using the rat Magnetic Luminex Performance Assay multiplex kit (R & D Systems, Minneapolis, MN, USA). Colon and hippocampus tissues were homogenized in a lysis buffer containing 20 mM Tris-HCl, 1 mM PMSF, 0.05% Tween-20 and a 1 × protease/phosphatase inhibitor cocktail (Cell Signaling Technology, Beverly, MA, USA). The protein concentrations in tissues were assessed using the BCA assay kit (Thermo Fisher Scientific). Tissue cytokine levels were normalized to the total protein level. A MAGPIX (Luminex, Austin, TX, USA) was used for the reading of the multiplex assay. All samples were assayed in duplicate, and the means of the duplicate values are represented.

### 4.6. Kynurenine and Tryptophan Assay

Kynurenine and tryptophan levels were measured in the serum and the hippocampus using a kynurenine enzyme-linked immunosorbent assay (ELISA) kit (MyBioSource, San Diego, CA, USA) and tryptophan ELISA kit (ImmuSmol, Bordeaux, France) according to the manufacturers’ instructions. The hippocampus was homogenized and prepared by the same protocol with cytokine measurements. The hippocampal kynurenine and tryptophan levels were normalized to the total protein levels. The absorbance (450 nm) was measured with a microplate reader (Molecular Devices, San José, CA, USA). All samples were assayed in duplicate, and the mean of the duplicate values are represented.

### 4.7. Quantitative Real-Time PCR (qRT-PCR)

Total RNA was extracted from the distal colon and hippocampus using an RNeasy Mini kit (Qiagen). cDNA was synthesized from the total RNA using a 1st-Strand cDNA Synthesis Kit (BioAssay Co, Daejeon, Korea) according to the manufacturer’s instructions. qRT-PCR was performed using a Real-Time PCR EvaGreen Kit (SolGent, Daejeon, Korea) and specific primers for each gene (Table S1) in a StepOnePlus Real-Time PCR System (Applied Biosystems Inc., Carlsbad, CA, USA). In order to confirm the amplification specificity of the PCR products, melting curve analysis was carried out after the completion of PCR cycling. The relative quantification of the mRNA transcripts was calculated based on the 2^−ΔΔCT^ method [74]. The amounts of each mRNA were estimated by normalization to the mRNA expression of Gapdh and Ubc for colon and brain samples, respectively. All samples were assayed in duplicate, and the means of the duplicate values are represented.

### 4.8. Western Blot Analysis

The distal colon and hippocampus isolated from each rat were homogenized using the lysis buffer described above for the cytokine measurements. The protein concentration was assessed using the Bradford reagent (Sigma-Aldrich, St. Louis, MO, USA). Equal amounts of proteins (50 µg) were subjected to sodium dodecyl sulfate (SDS)-polyacrylamide gels electrophoresis, and the electrophoresed proteins were transferred onto a nitrocellulose membrane (Amersham Biosciences, Uppsala, Sweden). The membranes were incubated with 5% skim milk and then with rabbit Zo-1 (Thermo Fisher Scientific), rabbit Ido (Biorbyt Ltd., Cambridge, UK) and rabbit Tdo-2 (MyBioSource) primary antibodies at 4 °C overnight. Then, the membranes were incubated with horseradish peroxidase-conjugated anti-rabbit IgG antibody (GeneTex, Irvine, CA, USA). The protein bands were visualized using the enhanced chemiluminescence (ECL) substrate (Bio-Rad Laboratories, Hercules, CA, USA). Band intensities were quantified using the ImageJ image analysis software (v. 1.4; NIH).

### 4.9. Statistical Analysis

The results are expressed as means ± standard errors of the mean (SEMs). Statistical analysis was carried out, using IBM SPSS Statistics 23 (SPSS Inc., Chicago, IL, USA). Data were assessed for the normality of variable distribution by the Shapiro–Wilk test. Data for the multiplex cytokine measurement were not following a normal distribution. Thus, the Wilcoxon rank-sum test was carried out for the multiplex cytokine measurement. The FDR *p*-value adjustment method was used to address the multiple-comparison issue using the Benjamini–Hochberg algorithm. Data for KYN and TRP assay, qRT-PCR and Western blot analysis showed a normal variable distribution with the homogeneity of variances (verified with the Levene test). A two-way ANOVA was performed for the KYN and TRP assay, qRT-PCR and Western blot analysis. The sample size and effect sizes were estimated using G*Power 3 [75]. Values of *p* < 0.05 were considered as statistically significant.

## Figures and Tables

**Table 1:** Cytokine levels in the serum, colon and hippocampus of MS rat.

Cytokine	Male		Female	
	Con	MS	Con	MS
Serum (pg/mL)				
IFN-γ	-	-	-	-
IL-1α	-	-	-	-
IL-1β	**2.7 ± 0.6**	5.8 ± 0.9 *^,#^	1.1 ± 0.4	-
IL-2	10.9 ± 1.3	10.0 ± 0.3	8.5 ± 1.4	6.4 ± 1.0
IL-4	-	-	-	-
IL-6	30.8 ± 4.4	23.1 ± 3.9	10.2 ± 2.6	12.2 ± 2.9
IL-10	-	-	-	-
IL-13	-	-	-	-
Colon (pg/mg of protein)				
IFN-γ	**23.9 ± 3.2**	57.4 ± 5.7 *^,#^	61.2 ± 7.3	55.2 ± 15.7
IL-1α	4.0 ± 0.9	4.8 ± 1.3	4.3 ± 1.0	4.9 ± 1.1
IL-1β	55.5 ± 8.1	61.9 ± 6.8	80.7 ± 3.3	67.3 ± 3.0 *
IL-2	2.6 ± 0.4	3.6 ± 0.5	3.2 ± 0.4	2.5 ± 0.3
IL-4	0.4 ± 0.1	0.7 ± 0.2	0.6 ± 0.2	0.6 ± 0.2
IL-6	**15.8 ± 0.8**	22.0 ± 1.7 *^,#^	19.1 ± 0.7	17.9 ± 1.0
IL-10	-	-	-	-
IL-13	-	-	-	-
Hippocampus (pg/mg of protein)				
IFN-γ	-	-	-	-
IL-1α	0.8 ± 0.3	0.7± 0.5	0.6 ± 0.3	-
IL-1β	**2.3 ± 0.1**	3.2 ± 0.3 *^,#^	2.3 ± 0.2	3.3 ± 0.5 *
IL-2	1.1 ± 0.0	0.9 ± 0.0	0.4 ± 0.0	0.6 ± 0.1 *
IL-4	**0.5 ± 0.1**	0.2 ± 0.0 *^,#^	0.1 ± 0.0	0.2 ± 0.1
IL-6	13.6 ± 1.0	15.0 ± 1.1	10.7 ± 0.8	13.8 ± 1.7
IL-10	-	-	-	-
IL-13	-	-	-	-

All data are presented as mean ± SEM. The Wilcoxon rank-sum test was performed and the *p*-values of <0.05 were considered statistically significant. * *p* < 0.05, ^#^ adjusted *p* < 0.05, *n* = 10 per group. Bold character means cytokines showing statistically different between control and MS rats after the adjustment using the Benjamini–Hochberg algorithm.

## Supplementary Materials

The following are available online at https://www.mdpi.com/1422-0067/22/4/1899/s1, Figure S1: The mRNA expression of occludin (Ocln) and claudin (Cldn) 1-5 genes in the colon and hippocampus of maternal separation (MS) rats. Figure S2: The mRNA expression of kynurenine 3-monooxygenase (Kmo) and kynurenine aminotransferase (Kat) in the hippocampus of maternal separation (MS) rats. Table S1: Primer sequences used for qRT-PCR.
