# Supplementary material for: Early-Life Stress Modulates Gut Microbiota and Peripheral and Central Inflammation in a Sex-Dependent Manner
Source: Int J Mol Sci. 2021 Feb 14;22(4):1899. doi: 10.3390/ijms22041899 (PMC7918891; doi:10.3390/ijms22041899)
Supplement: Supplementary file 1 [file ijms-22-01899-s001.pdf]

International Journal of
Molecular Sciences

MDPI

Table S1. Primer sequences used for qRT-PCR.

Gene		5'→3'
Ido-1	Sense	TTAAGACGCAATGAAGACT
	Anti-sense	GAGGTGGAACATTCTGAG
Ido-2	Sense	ACGACGACGATGATGATA
	Anti-sense	GGCTGAAGGATAGTAAGATAC
Tdo-1	Sense	TTCCAGAGTCTTCAGTTC
	Anti-sense	CTCATTGTAGTCTCCTTCA
Zo-1	Sense	GCATTATTCGCCTTCATACAA
	Anti-sense	ACACAACAATCGGATACCA
Gapdh	Sense	AGTGGCAAAGTGGAGATT
	Anti-sense	GTGGAGTCATACTGGAACA
Ubc	Sense	AGAAGGTCAAACAGGAAGATA
	Anti-sense	CCAAGAACAAGCACAAGAA

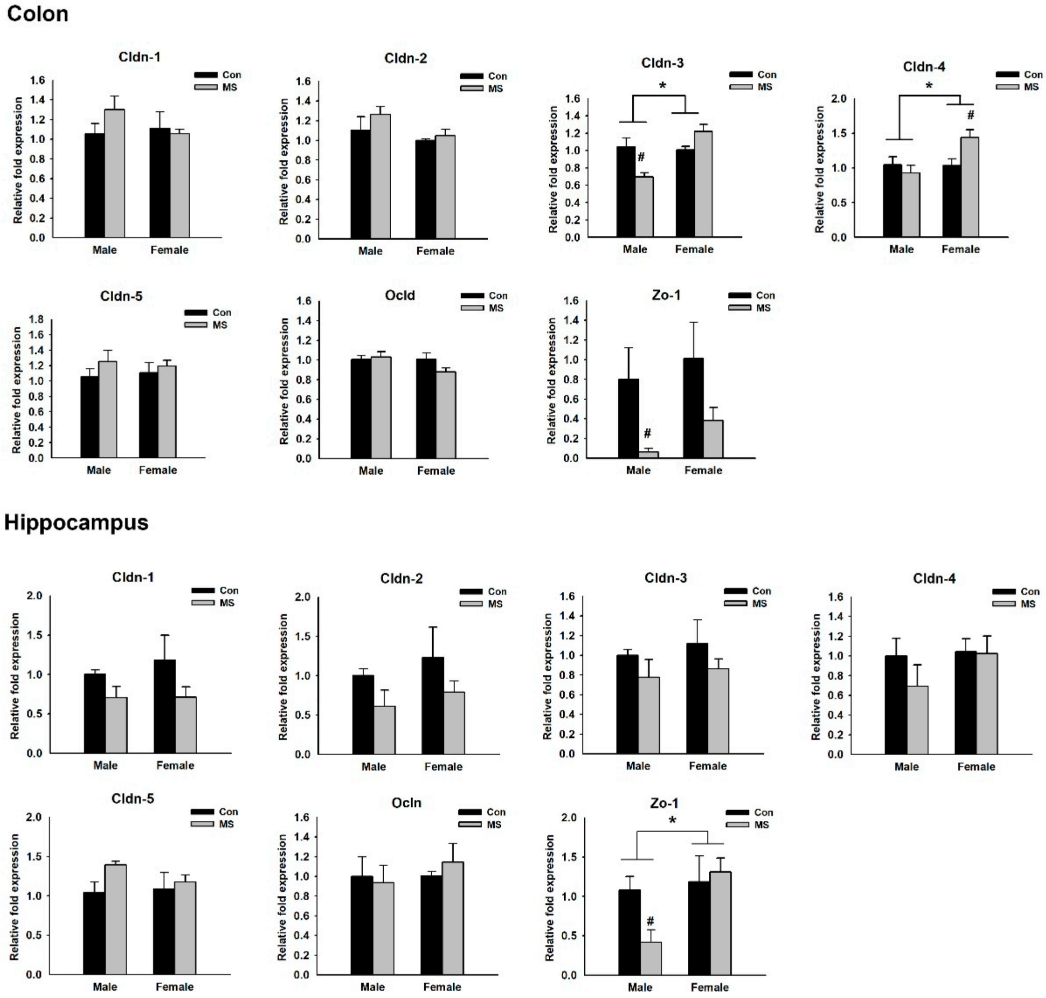

Figure S1. The mRNA expression of occludin (Ocln) and claudin (Cldn) 1-5 genes in the colon and hippocampus of maternal separation (MS) rats. The mRNA expression was detected through qRT-PCR using specific primers. Data are expressed as mean ± SEM. A two-way ANOVA test, * $p < 0.05$ on male vs. female; # $p < 0.05$ on control vs. MS within each sex. $n = 10$ per group in the colon, $n = 6$ per group in the hippocampus.

Cldn1, 5′-AGACCAGACTTCCTTACGA-3′; 5′-TAGAATCAAGCCTCAGAA-TAACA-3′

Cldn2, 5′-TTCATCCTTGGTGGTATC-3′; 5′-CAGTGGTGAGTAGAAGTC-3′

Cldn3, 5′-CCACACGCACACACATAT-3′; 5′-CCCAACCCTCTTCACCAT-3′

Cldn4, 5′-GCCAGCAACTATGTGTAAG-3′; 5′-GCCGTTATGAGTTCAATCC-3′

Cldn5, 5′-GCTCAGAACAGACTACAG-3′; 5′-AATCTTAGAGTTCCACCAT-3′

Ocld, 5′-TTGCTTCATCGCTTCCTT-3′; 5′-TCAAGTAGTATCTTCTCGTTCTG-3′

ZO-1, 5′-GCATTATTCGCCTTCATACAA-3′; 5′-ACACAACAATCGGATACCA-3′

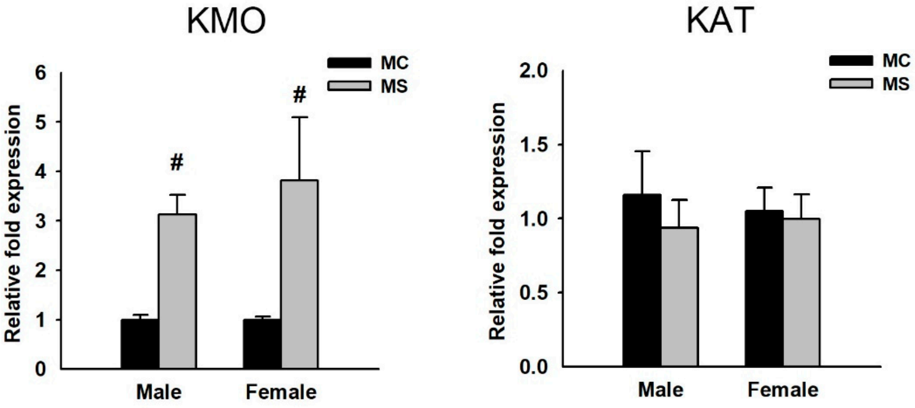

Figure S2. The mRNA expression of kynurenine 3-monooxygenase (Kmo) and kynurenine aminotransferase (Kat) in the hippocampus of maternal separation (MS) rats. The mRNA expression was detected through qRT-PCR using specific primers (Kmo, 5′-TTGAAGACTGCTTGGTATT-3′ and 5′-GCTCGCATCTCTATGTAAT-3′; Kat, 5′-CTTGGATTATAGGA-GATGAGAG-3′ and 5′-GAATGATAACAGCAGGAGAT-3′). Data are expressed as mean ± SEM. A two-way ANOVA test, * $p < 0.05$ on male vs. female; # $p < 0.05$ on control vs. MS within each sex. $n = 6$ per group.
